# BCG Vaccination and the Risk of Type 1 Diabetes Mellitus: A Systematic Review and Meta-Analysis

**DOI:** 10.3390/pathogens12040581

**Published:** 2023-04-12

**Authors:** Parnian Jamshidi, Bardia Danaei, Benyamin Mohammadzadeh, Mahta Arbabi, Amirhossein Nayebzade, Leonardo A. Sechi, Mohammad Javad Nasiri

**Affiliations:** 1 Department of Microbiology, School of Medicine, Shahid Beheshti University of Medical Sciences, Tehran 1985717443, Iran; parnian.jamshidi7@gmail.com (P.J.);; 2 Center of Public Health, Environmental and Occupational Hazards Control, Shahid Beheshti University of Medical Science, Tehran 1985717443, Iran; 3 School of Medicine, Shahid Beheshti University of Medical Sciences, Tehran 1985717443, Iran; 4 Department of Biomedical Sciences, University of Sassari, 07100 Sassari, Italy; 5 SC Microbiologia e Virologia, Azienda Ospedaliera Universitaria, 07100 Sassari, Italy

**Keywords:** type 1 diabetes, BCG vaccine, glycated hemoglobin A, autoimmunity, insulin, Bacillus Calmette Guerin vaccine

## Abstract

(1) Background: Type 1 diabetes mellitus (T1D) is an autoimmune disease characterized by progressive and irreversible autoimmune destruction of pancreatic beta cell islets, resulting in absolute insulin deficiency. To date, several epidemiologic and observational studies have evaluated the possible impact of BCG vaccination on T1D development, but the results are controversial. To elucidate this issue, we aimed to conduct a systematic review and meta-analysis of published cohort studies in this field. (2) Methods: A systematic search was performed for relevant studies published up to 20 September 2022 using Pubmed/Medline, Embase, and Scopus. Cohort studies, containing original information about the association between T1D and BCG vaccination, were included for further analysis. Pooled estimates and 95% confidence intervals (CI) for the risk ratio of T1D in BCG-vaccinated individuals compared to unvaccinated ones were assessed using the fixed effect model. (3) Results: Out of 630 potentially relevant articles, five cohort studies met the inclusion criteria. The total population of all included studies was 864,582. The overall pooled risk ratio of T1D development in BCG vaccinated and unvaccinated individuals was found to be 1.018 (95% CI 0.908–1.141, I^2^: 0%). (4) Conclusions: Our study revealed no protective or facilitative effect of prior BCG vaccination in T1D development.

## 1. Introduction

Type 1 diabetes mellitus (T1D) is an autoimmune disease characterized by progressive and irreversible autoimmune destruction of pancreatic beta cell islets, resulting in absolute insulin deficiency. The incidence rate of T1D has been increasing globally by 2–3% yearly [1]. In 2021, there were about 8.4 million affected individuals worldwide, and, of these, 1.5 million (18%) were younger than 20 years of age [2]. Therefore, finding a manner to prevent T1D is of great public health significance.

It seems that, in genetically susceptible individuals, some environmental factors trigger the autoimmune process, leading to T1D [3]. To date, several environmental factors have been investigated in this regard, including pre- and postnatal infections, especially viral infections, vitamin supplementation, early infant diet, and vaccinations [4,5,6,7,8].

The Bacillus-Calmette-Guérin (BCG) vaccine, extracted from the attenuated strain of *Mycobacterium bovis*, is one of the most common vaccines worldwide [9]. It has been used since 1920 to immunize infants, children, and adults against tuberculosis with limited side effects [10]. Recent evidence suggests a non-specific impact of the BCG vaccine on the immune system, which gives hope to be a potential agent for the prevention or treatment of autoimmune diseases, such as T1D [11]. It modulates the immune system via the induction of cell-mediated immunity, leading to Th1, Th17, and T-reg cell stimulation. These cells are shown to be part of the autoimmune process of T1D [12,13,14]. However, the long-term effects of BCG and the necessary boosters for an appropriate T-reg response have not yet been investigated.

To date, several epidemiologic and observational studies have evaluated the possible impact of BCG vaccination on T1D development, but the results are controversial. To elucidate this issue, we aimed to conduct a systematic review and meta-analysis of published cohort studies in this field. To our knowledge, this is the first meta-analysis of cohort studies assessing the preventive role of BCG vaccination in developing T1D.

## 2. Methods

This review conforms to the “Preferred Reporting Items for Systematic Reviews and Meta-Analyses” (PRISMA) statement [15]. Registration: PROSPERO (pending registration ID: 377509).

### 2.1. Search Strategy

A systematic search was performed for relevant studies published up to 20 September 2022 using Pubmed/Medline, Embase, and Scopus.

Articles with the following keywords in titles or abstracts were selected: “Type 1 Diabetes Mellitus” OR “Insulin Dependent Diabetes Mellitus” OR “Juvenile Onset Diabetes” OR “Sudden Onset Diabetes Mellitus” OR “Type 1 Diabetes” OR “Brittle Diabetes Mellitus” OR “Ketosis-Prone Diabetes Mellitus” OR “Autoimmune Diabetes” AND “BCG Vaccine” OR “Bacillus Calmette Guerin Vaccine” OR “Calmette Guerin Bacillus Vaccine” OR “Calmette Vaccine”.

### 2.2. Study Selection

The records found through database searching were merged, and the duplicates were removed using EndNote X8 (Thomson Reuters, New York, NY, USA). Two reviewers (BD and BM) independently conducted title and abstract screening of the records to exclude irrelevant studies. The full text of potentially eligible records was retrieved and evaluated by two other reviewers (PJ and MA). Cohort studies containing original information about the association between T1D and BCG vaccination were included for further analysis. Review articles, duplicate publications, conference abstracts, letters, animal studies, in vitro/in vivo studies, and those with insufficient data were excluded from the analysis. No language restrictions were considered.

### 2.3. Data Extraction

A data extraction form was designed by PJ, and the following data were extracted: first author’s name, type of study, year of publication, time of the study, the country where the study was conducted, study population, population risk factors, mean age of the total study population, gender, mean age at BCG vaccination, number of BCG vaccination, mean age at T1D diagnosis, T1D diagnosis criteria, follow-up duration and technique, immunologic modifications, the association between BCG vaccination and T1D, and type of association, as well as the total number of vaccinated and unvaccinated individuals, number of vaccinated cases with and without T1D, and number of unvaccinated cases with and without T1D. Selected data were extracted from the full texts of eligible publications by BD, BM, and PJ.

### 2.4. Quality Assessment

The Newcastle-Ottawa Scale (NOS) for cohort studies was used to perform a quality assessment of the included studies [16]. The NOS scale evaluates the risk of bias in observational studies with three areas: (1) selection of participants, (2) comparability, and (3) outcomes. A study can be given a maximum of one point for each numbered item within the selection and outcome domains, and a maximum of two points can be assigned for comparability. Scores of 0–3, 4–6, and 7–9 were allocated for the low, moderate, and high-quality studies, respectively.

### 2.5. Meta-Analysis

Statistical analyses were performed with the Comprehensive Meta-Analysis software, version 2.0 (Biostat Inc., Englewood, NJ, USA). Pooled estimates and 95% confidence intervals (CI) for the risk ratio of T1D in BCG-vaccinated individuals compared to unvaccinated ones were assessed. The fixed-effects model was used due to the estimated heterogeneity of the true effect sizes. The between-study heterogeneity was assessed by Cochran’s Q and the I^2^ statistic. Publication bias was evaluated statistically using Begg’s tests (*p* < 0.05 was considered indicative of statistically significant publication bias).

## 3. Results

**Figure 1:**
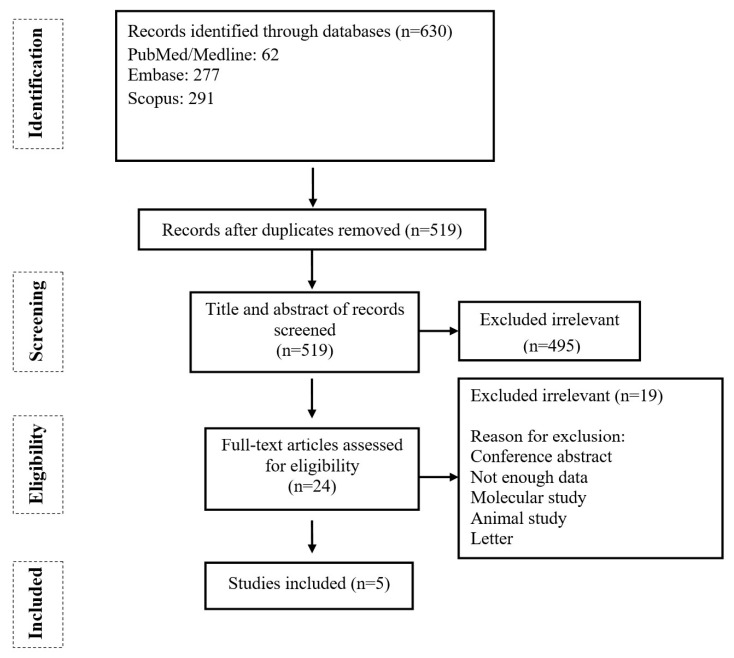
Flow chart of study selection for inclusion in the systematic review and meta-analysis.

The primary search identified a total of 630 potentially relevant publications. After removing the duplicates, 519 studies were considered for the title and abstract screening, of which 495 irrelevant articles were excluded, and 24 papers were selected for full-text assessment. Finally, five cohort studies met the inclusion criteria and were considered for further meta-analysis (Figure 1).

### 3.1. Quality of Included Studies

Based on the Newcastle-Ottawa Scale, which was used to assess the quality of the cohort studies, the mean (standard deviation [SD]) NOS score was 9 (0.0), which is suggestive of a high methodological quality and a low risk-of-bias of the included studies (Table 1).

### 3.2. Characteristics of the Included Studies and Populations

The study period ranged from 1974 to 2014. Most of the studies were conducted in Canada (*n* = 3), and the rest were conducted in Germany (*n* = 2) (Table 2). The total population of all included studies was 864,582 (Table 2), of which, with the available data concerning gender, 48.76% (*n* = 420,474) of participants were female, and 51.23% (*n* = 441,835) of participants were male. All the subjects of the two studies were newborns, and the mean age of other individuals with available data was 18.5 years (Table 3). Two studies considered diabetic parents as their population risk factors. Most of the individuals were vaccinated in the first year of their life (90%, *n* = 390,365), while others were vaccinated after the first year of age (9.99%, *n* = 43,333) or after three months of age (0.002%, *n* = 10). An amount of 94.46% of our studied population were given only one dose of the BCG vaccine (*n* = 367,956), and 5.54% of individuals were vaccinated with two doses or more of the BCG vaccine (*n* = 21,575). Our cohort populations were followed up for a duration of nine months to 18 years in different studies (Table 3). Various T1D diagnosis and follow-up techniques were used to investigate the effect of the BCG vaccine on the incidence or progression of T1D. For more details, see Table 2 and Table 3.

Only one study confirmed the association between type 1 diabetes and BCG vaccination. It reported that BCG vaccination before one year of age had a preventive role after 30 years old. Four other studies refused the preventive role of BCG vaccination in T1D (Table 4).

Two studies evaluated the immunological markers in their study cohort. Islet cell antibodies increased in one study; however, in another study, they remained unchanged. Anti-glutamic acid decarboxylase antibodies and insulinoma-associated protein-2 were also unchanged in one study (Table 4).

### 3.3. Meta-Analysis

**Figure 2:**
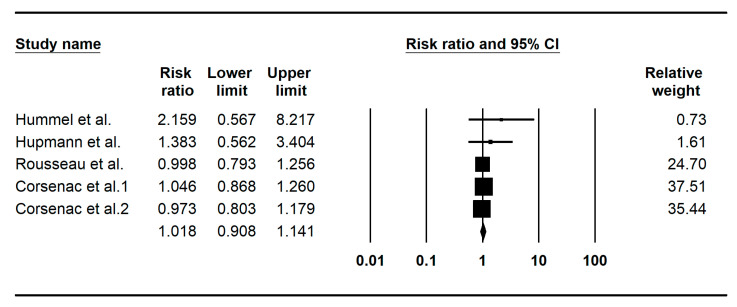
Forest plot of the cohort studies indicating the impact of prior BCG vaccination in T1D development. Hummel et al. [17]; Hupmann et al. [18]; Rousseau et al. [10]; Corsenac et al. [9]; Corsenac et al. [19].

The overall pooled risk ratio of T1D development in BCG vaccinated and unvaccinated individuals was found to be 1.018 (95% CI 0.908–1.141, I^2^: 0%), indicating no impact of BCG vaccination in T1D development (Figure 2). There was no evidence of publication bias (Begg’s test *p* > 0.05).

## 4. Discussion

To our knowledge, this is the first systematic review and meta-analysis of cohort studies evaluating the effect of the prior BCG vaccination in the occurrence of type 1 diabetes (T1D). Despite the existence of mechanisms justifying the protective role of BCG vaccination in T1D development, our meta-analysis did not show any decreased risk, which concurs with Chang et al.’s meta-analysis study of randomized controlled trials [20].

Different mechanisms, involving the innate and adaptive immune system, have been suggested for the protective or therapeutic role of the BCG vaccine in autoimmune disorders, such as T1D. In the T1D process of autoimmunity, the imbalance between T-reg cells and cytotoxic T lymphocytes (CTLs) leads to the destruction of pancreatic islet cells [21]. Both BCG vaccination and tuberculosis infection lead to tumor necrosis factor (TNF) production that results in the activation of T-reg cells and the reduction of CTLs [22,23,24,25,26,27]. Evidence suggests the role of epigenetic changes in some signature genes of T-reg, leading to T-reg activation. Multi-dose BCG vaccinations result in the demethylation of T-reg key signature genes. It is shown that one of these critical genes is FOXP3, which is over-methylated in diabetic patients, and 22 CpG sites of the FOXP3 gene become demethylated after three years of BCG therapy [28,29]. Furthermore, the BCG vaccine affects innate immune responses by causing persistent changes in the host’s monocytes; it is controlled by the histone activity of key genes involved in cytokine secretion [30,31]. Animal studies on diabetic mice have shown the BCG vaccine’s efficacy in regenerating pancreatic beta cells [32]. Additionally, a suppressing effect of the BCG vaccine through macrophages has been demonstrated against various lymphocyte functions [33].

After BCG vaccination, a metabolic switch from overactive oxidative phosphorylation to aerobic glycolysis is induced in immune system cells. As a result, the following effects occur: increase in early glycolytic enzymes, up-regulation of glucose uptake that results in lowering of blood glucose, shunting of the accelerated glucose utilization through the pentose phosphate pathway, decrease in utilization of the late glycolytic steps, including the Krebs cycle, increase in lactate production, and decrease in oxidative phosphorylation [27,34,35,36,37,38].

Regarding the mechanisms mentioned above, this vaccine seems to have some immune-modulatory properties. Still, our data suggest that, concerning T1D, these properties cannot prevent disease development in susceptible individuals. However, in the cohort study conducted by Corsenac et al., there were some protective effects after 30 years in participants who were vaccinated before one year of age [19]. Most of our reviewed studies did not follow the cases up to more than 30 years of age. Hence, it is not apparent whether our result is due to the delayed effect of the BCG vaccine that is not well evaluated yet, or whether there is not any protective effect of BCG in T1D development at any age. Further investigation with long-term follow-up is necessary in this regard. Doupis et al. showed protective effects of BCG vaccination in the form of delaying T1D onset in their retrospective observational study [22]. This result contrasts with a study conducted in Germany by Huppmann et al., revealing that neonatal BCG vaccination may play a role in the acceleration of progression from autoimmunity to diabetes, probably because of the powerful immunostimulatory effect of BCG, as manifested by the enlargement of lymph nodes by the site of injection [18]. It was shown that BCG can enhance the activities of CD4+ Th1 cells and CD8+ T cells, which have cytotoxic properties by promoting antigen presentation [39,40]. This can suggest that, in some manner, BCG vaccination can promote immune responses, causing acceleration in auto-immune disease development in susceptible individuals. However, this has not been supported by rational evidence.

Another critical point is that the immune system response contains humoral immunity, which produces auto-antibodies that may precede T1D’s clinical manifestation onset for years. The main autoantibodies detected in patients with T1DM are those against GAD65, zinc transporter (ZnT8), tyrosyl phosphatase (IA-2), and insulin (IAA) [41]. However, the humoral immune response to BCG vaccination and this vaccine’s effect on the humoral arm of the immune system remains poorly defined, and there is variable evidence for the induction and regulation of specific antibody responses [42]. In our reviewed studies, Hummel et al. reported an increase in islet cell antibodies in BCG-vaccinated individuals [17]. In contrast, Hupmann et al. showed that BCG vaccination did not affect autoantibody development [18].

Growing evidence supports the involvement of *Mycobacterium avium* subspecies *paratuberculosis* (MAP) in T1D pathogenesis [43,44,45]. Several MAP antigens, including heat shock protein 65 (Hsp65) and MAP3865c, have been identified to have relevant sequence homology with GAD65 and ZnT8, expressed by pancreatic beta cells, respectively [46,47,48,49,50,51]. As a result, MAP infectivity may trigger the autoimmunity process, leading to T1D. Since BCG and MAP have common antigens (e.g., Hsp65) [52,53], BCG vaccination might partially protect from developing T1D, probably due to a mitigating action upon MAP [46]. However, the BCG vaccine is the attenuated form of *M. bovis*, a different species from MAP, and our conclusion does not rule out the potential of MAP to cause T1D. Further investigations are required to clarify this issue.

Previously, the duration of BCG-induced protection has been considered limited to the first few years of life. However, recent studies revealed that BCG is effective for at least 20 years against tuberculosis (TB) when given at birth or school ages [54,55]. Additionally, some evidence reveals that BCG-induced protection becomes weaker as time passes, despite its long-lasting effect [56]. This can suggest that the BCG vaccine’s immune-modulatory properties may also weaken through the years, hence losing its protective effect against auto-immune diseases, such as T1D.

BCG vaccine induces regional inflammatory responses with granulocyte influx in the preliminary phase, followed by CD4+ T-cells, as well as rising levels of pro-inflammatory Th1-type cytokines, i.e., IL-2, IL-12, and IFN-γ [57]. In the studies related to cancer treatment with the help of the BCG vaccine (i.e., bladder cancer and melanoma), laboratory and clinical evidence shows that the anti-tumor activity is focused on the site of BCG administration, which supports the view that the effects of BCG on the immune system are prominently local [40,58]. The distance between the site of the desired outcome (beta cells in the pancreas) and the BCG injection site, as well as the long-time interval between the time of vaccine injection and the time of disease in some individuals, can explain the ineffectiveness of this vaccine in prevention of T1D.

In terms of sex difference’s impact on BCG vaccination outcome, Koeken et al. found a significant sex-differential effect, with non-negligible reductions in inflammatory proteins after BCG administration in males only [59]. This was supported by another study by Rhodes et al., which showed a weak association between a higher baseline response and being male [60]. However, in the included studies in our systematic review, when comparing the sex effect modification, there were no significant differences between participants. Furthermore, BCG vaccination may have different impacts according to age at vaccination. Evidence suggests that the infancy period is of great importance in the maturation of the immune system [61]. Vaccination in the first year of life is associated with better heterologous innate stimulation and proinflammatory cytokine/chemokine production [62,63]. In our study, 90% of the individuals were vaccinated in the first year of life, suggestive of the best performance of the BCG vaccine. It is necessary to mention that, according to insufficient classified data given by the included studies in our meta-analysis, we could not perform any subgroup analysis regarding the age at vaccination and sex of the individuals.

In the studies included in our review, there was no comparison between different administered BCG strains. In some studies, it is manifested that, from those who were vaccinated with varying BCG vaccine strains, different degrees of T-cell responses are induced in in vitro peripheral blood mononuclear cell (PBMC) cultures [64,65]. However, there is conflicting information with no confirmed evidence supporting the variability in efficacy or the protection given by different BCG vaccine strains, and further investigations are warranted [66].

In our study, the vast majority (94.46%) of the vaccinated population received only a single dose of the BCG vaccine, along with the standard required regimen for the prevention of TB [20]. However, several animal and human studies suggest the application of multiple doses of BCG to achieve a protective effect against T1D [22,67]. Therefore, we cannot ignore this probable effect as a potential bias for our conclusion. Further investigations are needed to compare the single and multiple doses of BCG in T1D development.

## 5. Limitations

Our study has some limitations. First, the scarcity of cohort studies and the probability of overlap between studied populations is the major limitation of our study. Three out of our five included studies were from Canada, and the other two were from Germany. Despite the differences in age groups, study periods, and the number of cohort populations of each study, there might probably be some overlap between study populations in articles published from the same country. We could not confirm or ignore this issue. However, it will challenge our study conclusion, and further cohort studies from different countries are greatly required to make any solid conclusion in this regard. Secondly, although our included studies for meta-analysis were cohort studies with large samples and long follow-up durations, most did not follow the cases until adulthood. This may conceal the effect of vaccination at older ages. Thirdly, 94.46% of our vaccinated cases received a unique dose of BCG, and the impact of multiple dosing vaccinations has remained unclear. Fourthly, due to the unavailable data of our included studies, we could not conduct any subgroup meta-analysis to evaluate the effects of age, sex, age at vaccination, immunization dose, etc. in conclusion. Fifthly, most of our reviewed studies complained about the lack of adjustment for potential confounders, which may affect our analysis. Sixthly, different studies conform to varying criteria for T1D diagnosis of their cases. Additionally, they used different follow-up techniques, as the studies from Germany only considered autoimmunity and immunologic modifications among their cohort patients, while the studies from Canada followed clinical improvements in their patients. These issues may lead to heterogeneity for a definite conclusion. Seventhly, some of our included studies used self-reported data instead of registered information about BCG vaccination status, which has poor validity and may conform to recall and misclassification bias.

## 6. Conclusions

Based on our study, it seems that there is no protective or facilitative effect of prior BCG vaccination in T1D development. Although some studies showed that BCG vaccination causes a delay in T1D occurrence or has a late outgrowth in adulthood, these studies are limited. There is little evidence on this topic, and more extensive observational studies with long-term follow-up duration are required to delineate these issues. Additionally, further studies are warranted to compare the effect of multiple dosing vaccination and different time of exposure on the possible preventive role of BCG in T1D development.

## Figures and Tables

**Table 1:** Quality assessment of the observational studies included in the meta-analysis (The NOS tool).

Author	Selection				Comparability		Outcome			Total Quality Score
	Representativeness of Exposed Cohort	Selection of Non-Exposed Cohort	Ascertainment of Exposure	Demonstration That Outcome of Interest Was Not Present at the Start of the Study	Adjust for the Most Important Risk Factors	Adjust for Other Risk Factors	Assessment of Outcome	Follow-Up Length	Loss to Follow-Up Rate	
Hummel et al. [17]	1	1	1	1	1	1	1	1	1	9
Hupmann et al. [18]	1	1	1	1	1	1	1	1	1	9
Rousseau et al. [10]	1	1	1	1	1	1	1	1	1	9
Corsenac et al. [9]	1	1	1	1	1	1	1	1	1	9
Corsenac et al. [19]	1	1	1	1	1	1	1	1	1	9

NOS: The Newcastle-Ottawa Scale.

**Table 2:** Characteristics of the included studies.

Authors	Type of Study	Year of Publication	Time of the Study	Country	Study Population	T1D Diagnosis Criteria
Hummel et al. [17]	Prospective Cohort	2000	1989–1999	Germany	658	NA
Hupmann et al. [18]	Prospective Cohort (Brief Report)	2005	1989–2000	Germany	1610	World Health Organization criteria
Rousseau et al. [10]	Retrospective Cohort	2016	1974–1994	Canada	78,492	One of these definitions: (1) ≥2 diabetes-related medical visits within 2 years or ≥1 hospitalization for diabetes (2) ≥4 diabetes-related medical visits within 2 years
Corsenac et al. [9]	Prospective Cohort	2021	1985–1993	Canada	387,704	≥4 related physician claims over a 2-years period, with at least 30 days between two claims
Corsenac et al. [19]	Prospective Cohort	2021	1997–2014	Canada	396,118	≥1 hospitalization OR ≥2 physician claims for DM within two years, with ≥1 day between two services AND using only insulin

NA: Not available.

**Table 3:** Characteristics of the study population.

Authors	Age (Mean)	Gender	Population Risk Factors	Age at BCG Vaccination (Mean)	Number of BCG Vaccination	Age at T1D Diagnosis (Mean)	Follow-Up Duration	Follow-Up Technique
Hummel et al. [17]	Newborn	NA	Diabetic parents	<6 weeks	1 dose	3.85 years	2 years	Antibody assays (antibodies to islet cells (ICAs), insulin (IAAs), GAD (GADAs), and IA-2 (IA-2As), HLA determination (HLA class II typing), Questionnaire
Hupmann et al. [18]	Newborn	NA	Diabetic parents (T1D)	<3 month: 206, >3 month: 10	1 dose	NA	Follow-up at nine months, and 2, 5, 8, and 11 years old	Measurement of Islet autoantibodies (antibodies to insulin, GAD, and insulinoma-associated protein-2)
Rousseau et al. [10]	NA	40128 M, 38359 F	NA	<1 year	1 dose	NA	NA	Diabetes-related medical visits
Corsenac et al. [9]	12.5 years	199380 M, 188324 F	NA	Newborns and school children (<1 year: 156,342, ≥1 year: 21,791)	1 dose: 167,274, ≥2 doses: 10,859	NA	9 years	Follow-up for T1D diagnosis: ≥4 related physician claims over a 2-year period, with at least 30 days between two claims
Corsenac et al. [19]	24.5 years	202327 M, 193791 F	NA	Newborns and school children (<1 year: 154,667, ≥1 year: 21,542)	1 dose: 165,493, ≥2 doses: 10,716	NA	18 years	Follow-up for T1D diagnosis: ≥1 hospitalization OR ≥2 physician claims for DM within two years, with ≥1 day between two services AND using only insulin

NA: Not available.

**Table 4:** Association between BCG vaccination and T1D.

Authors	Type of Study	Association between BCG Vaccination and T1D	Type of Association	Immunologic Modifications
Hummel et al. [17]	Prospective Cohort	No	Not Preventive	Islet cell antibodies ↑
Hupmann et al. [18]	Prospective Cohort (Brief Report)	No	Not Preventive (Even may accelerate the progression from autoimmunity to T1D)	Islet cell antibodies (ICAs) = Anti-glutamic acid decarboxylase antibodies (anti-GAD antibodies) = Insulinoma-associated protein-2 =
Rousseau et al. [10]	Retrospective Cohort	No	Not Preventive	NA
Corsenac et al. [9]	Prospective Cohort	No	Not Preventive	NA
Corsenac et al. [19]	Prospective Cohort	Yes	Preventive after 30 years old and in individuals who were vaccinated before 1 year of age (no association was found before 30 years old, but vaccinated subjects had a lower risk of this phenotype after age 30)	NA

NA: Not available; The use of “=” means not changed.

## Data Availability

All data were included in the manuscript.
